# Assessing Mental Illness Stigma: A Complex Issue

**DOI:** 10.3389/fpsyg.2018.02722

**Published:** 2019-01-11

**Authors:** Stefania Mannarini, Alessandro Rossi

**Affiliations:** ^1^Interdepartmental Center for Family Research, University of Padova, Padova, Italy; ^2^Section of Applied Psychology, Department of Philosophy, Sociology, Education, and Applied Psychology, University of Padova, Padova, Italy

**Keywords:** mental illness stigma, mental illness, stigma, mental health stigma, assessment

To date, stigma toward individuals with Mental Illness (MI) is a severe social problem as well as a heavy burden for affected people (Corrigan, 2005; Oexle and Corrigan, 2018). While public knowledge about physical diseases is usually seen as beneficial, knowledge about MI is often disregarded (Angermeyer and Dietrich, 2006). As a consequence, many persons suffering from common mental disorders may not receive appropriate (therapeutic) social support due to the community's lack of awareness (Jorm, 2000; Ratti et al., 2017). Indeed, although an increase in the number of individuals seeking psychological support has been registered worldwide (Fang et al., 2011; Picco et al., 2016), a significant number of people still avoid asking for help. Possible causes of this mismatch are the tendency of some persons to think that MI difficulties will resolve spontaneously (Sareen et al., 2007; Wilson and Deane, 2012) and/or their reluctance to bear the costs of therapies (Vogel and Wester, 2003; Castelnuovo et al., 2016) and/or they might even consider the possible long-term ineffectiveness of certain approaches (Harding and Zahniser, 1994; Parker and Fletcher, 2007; Beutler, 2009; Barlow et al., 2013; Lilienfeld et al., 2014; Sorgente et al., 2017; Jackson et al., 2018). Moreover, people avoid seeking professional help due to the fear of disclosing a diagnosis which involves stigma associated with MI (Hinshaw, 2007; Mannarini and Boffo, 2015).

Indeed, although the knowledge of MI has been improved (Kendler and Prescott, 2006; Schnyder et al., 2018), scientific literature showed that people usually perceive individuals with MI as dangerous (Angermeyer and Matschinger, 2005). Walker and Read (2002) showed that people's dominant impression on MI is that it leads to unpredictable behaviors and loss of control. Scientific literature demonstrated otherwise that the relationship between the majority of psychiatric disorders and violent behaviors does not exist, showing that attitudes toward people with MI are in most cases related to a biased prejudice and/or stereotype (Walker and Read, 2002; Angermeyer and Matschinger, 2005; McGinty et al., 2018). People with MI are often subjected to discriminative and stigmatizing behaviors (Wahl, 1999; Hinshaw, 2007) in the social context (Corrigan et al., 2012), inside their families and among friends (Wahl, 1999; Hinshaw, 2007). This constant exposure to stigmatizing attitudes has numerous negative effects, such as: lower quality of life, critical impediments in seeking professional help, treatment discontinuity, and higher dropout rates (Sirey et al., 2001; Hinshaw, 2007; Livingston and Boyd, 2010).

In addition, stigma toward people with MI is both a cross-diagnostic (Mannarini and Boffo, 2015) and a cross-cultural phenomenon (Read and Harré, 2001; Jorm and Griffiths, 2008; Abdullah and Brown, 2011; Mannarini et al., 2017, 2018). A study conducted in three countries (Germany, Russia, and Mongolia), involving two mental disorders (depression and schizophrenia), revealed comparable overall results regardless of strong differences in cultural backgrounds. At the same time, findings also suggested differences at the level of specific indicators, namely in the relation between mental disorders, etiological causal beliefs, and social distance toward people with MI (Dietrich et al., 2004). More in detail, people were more likely to attribute the etiology of both pathologies to psychological causes instead of biological ones. It was also found that the endorsement of biological causes as primary beliefs of mental disorders was associated with a greater social distance. On this subject, in several samples of Italian participants, Magliano et al. (2004) showed that the main cause of social distance and stigmatization was the diagnosis attributed to the patient. Schomerus et al. (2011) examined 17 studies, representative of several populations, regarding stigma toward people with MI. Results suggested that there is a significant effect linked to the cultural context influencing how people perceive mental disorders and stigmatize individuals with MI: they showed that these differences concerned variables such as blaming, dangerousness, emotional reactions, and desire of distance (Schomerus et al., 2006). The significant effect of cultural characteristics on the perception of specific variables regarding MI individuals was also confirmed by other studies (Abdullah and Brown, 2011; Angermeyer et al., 2011; Schomerus et al., 2011). According to these results, Pescosolido et al. (2010) found that causal beliefs, attitudes toward people with MI and cultural context were also associated. Furthermore, they found a positive correlation between the tendency to give a biological explanation of mental disorders and a higher recommendation of therapeutic treatment(s) (Pescosolido et al., 2010). Finally, Mannarini and colleagues extended previous findings by studying etiological beliefs in relation to perceived dangerousness, social closeness, and avoidance toward people with MI in Italian and Israeli students. Using a Latent Class approach they found an interaction effect of culture on the latent structure of variables able to describe attitudes toward MI (Mannarini et al., 2016, 2017, 2018).

Etiological beliefs of mental disorders have been extensively studied from this perspective. Results revealed that—regardless of cross-cultural context—the most recurrently indicated types of causes were both biological and psychosocial factors (Jorm, 2000; Sears et al., 2011). In an attempt to achieve representative results, the relationship between etiological beliefs and discriminating attitudes toward MI people has driven to a long-lasting debate in the field, leading to an encouraging research production worldwide (Read and Harré, 2001; Walker and Read, 2002; Rüsch et al., 2005; Corrigan, 2016). Indeed, the cause to which the MI condition is attributed (psycho-sociological vs. bio-genetic) has been considered to be one of the main factors underlying the stigmatizing processes (Feldman and Crandall, 2007). Consequently, etiological beliefs have been used as a promotional trigger to overcome stigma in several public health programs aimed to reduce discrimination toward people with MI (Corrigan et al., 2018; Morgan et al., 2018). The promotion of bio-genetic approach to etiological beliefs of MI has been considered as the most promising approach to reduce stigma; this is due to the higher association between this specific belief and perception of onset/offset controllability (Larkings and Brown, 2018). By promoting a medical approach, these campaigns strongly emphasized the endorsement of biogenetic causal models of MI by explicitly portraying mental disorders as medical conditions that should be treated with medical treatments: *i.e*., the “*mental illness is an illness like any other*” (Corrigan, 2000; Read et al., 2006; Schomerus et al., 2012). The attribution theory framework could explain this perspective by postulating that specific emotional, attitudinal and behavioral responses toward a person are generated by causal attributions over his/her behaviors (Weiner, 1995). Along this line, when causes of mental health problems are attributed to external factors, outside of individual control, such as biological and/or genetic causes, the reactions toward persons with MI should be less negative. Conversely, if the cause of mental disorders is attributed to an individual's character that is considered an internal cause, people would be less willing to interact with him/her (Corrigan, 2000). Thus, campaigns aimed to reduce ascriptions of responsibility and guilt toward the affected persons, since such causes are beyond the individual control. Therefore, by aiming to change the perception that people with mental disorders should be blamed for their troubles, they may lead to lower rejection attitudes in social contexts (Corrigan, 2000).

However, despite these theoretically-based premises, these campaigns sponsoring a bio-genetic cause of mental disorders did not produce the desired effect in reducing stigmatizing attitudes. Indeed, the result of these social interventions was a mixed and contradictory pattern of both negative and positive emotions and cognitions, such as: higher levels of negative stigma as well as a higher endorsement of professional MI treatments and MI scientific literacy (Angermeyer et al., 2011; Kvaale et al., 2013). Indeed, these strategies didn't consider that bio-genetic causal beliefs are associated to the perception that people with MI are considered dangerous, uncontrollable, and antisocial (Read and Law, 1999). Proclaiming a “*wrong”* brain functioning as a cause of mental disorders strongly increase the dangerousness perception of such individuals, due to the possible unawareness of their behaviors (Read and Harré, 2001; Dietrich et al., 2004; Angermeyer and Matschinger, 2005). These results suggested that encouraging biological models as explanation of mental disorders is not useful to reduce stigmatization as well as social distance (Walker and Read, 2002; Angermeyer et al., 2011). In order to decrease social isolation of people with MI, campaigns against MI stigma should provide more attention to deepen the differences in opinions and beliefs over different disorders. This way, more adequate educational programs and appropriate clinical interventions could be developed (Corrigan et al., 2001; Angermeyer and Dietrich, 2006; Gatta et al., 2017) as well as the affective and public rights of the patients (Magliano et al., 2004). Thus, a precise and in-deep understanding of prejudice as well as stereotypes toward mental health disorders is fundamental to improve the effectiveness of mental health campaigns designed to reduce MI stigma (Monteith and Pettit, 2011).

However, it has to be highlighted that research on MI stigma showed some limitations. First of all, this subject has been studied mainly focusing on studies with self-report measures (Link et al., 2004). Questionnaires are aimed to assess opinions and attitudes about behaviors of a person with mental disorders, about his/her relationships and the etiology of MI (Luty et al., 2006; Mannarini and Boffo, 2013). However, since certain opinions and attitudes can be considered discriminatory, consequently it is likely that respondents' answers are influenced by social desirability concerns (Stier and Hinshaw, 2007). Along these lines, research on MI stigma could progress by focusing on the role of implicit processes and developing measures able to assess implicit cognitions, attitudes, and causal beliefs expressed beyond the individual's conscious control (Mannarini and Boffo, 2014). Considering that both controlled deliberate responses and automatic implicit responses are involved in stigmatization processes (Dovidio et al., 1997; Reeder and Pryor, 2008), indirect measurement procedures, such as the Implicit Association Test (IAT) (Greenwald et al., 1998) or other speeded reaction-time tasks, could bypass these issues.

In addition, research on this topic, as well as the heterogeneity of findings, showed that studies on MI stigma usually focus only on one (or few) factor(s) composing this multi-structured construct. Moreover, these studies did not consider more than one mental disorder *per* time, in particular: schizophrenia, depression, and addiction disorders (Room, 2005; Peluso and Blay, 2009; Pescosolido et al., 2010; Schomerus et al., 2011). Otherwise, the measurements of MI stigma are conducted by referring to MI as a universal category including heterogeneous and different diagnostic categories, thus leading to a loss of focus over each mental disorder specificities (Angermeyer and Matschinger, 2005; Jorm et al., 2006; Mannarini and Boffo, 2015). Along these lines, a vignette approach could overcome this issue. According to DSM-V diagnostic criteria (American Psychiatric Association, 2013), a vignette is a brief sketch and/or an unlabeled story representing problems and symptoms of a specific mental disorder (Angermeyer and Matschinger, 2005; Jorm et al., 2006). In the vignette procedure proposed by Mannarini and Boffo (2015) any specific mental disorder is mentioned in any part of the text, and participants were asked to evaluate several variables, such as: the etiology of the person's problems depicted in the vignette as well as possible treatments, the degree of dangerousness and the desire of social distance from that person.

In conclusion, considering the abovementioned background and the myriad of findings pointed out by the scientific research, it seems that assessing stigma is a very complex issue. Indeed, it involves complex evaluation procedures able to define structures of interactive variables, such as etiological beliefs, attitudes, prejudices, personal, and social problems, both toward mentally ill persons and in the mental disorders' perceiver, while taking into account the role of different cultures. A key role is also played by treatments and their relations with causal beliefs and with other variables. Furthermore, and of fundamental importance in this context is the distinction of different mental disorders, such as schizophrenia, depression, and addiction, which should be studied separately to highlight the specificity of their relations with stigma.

## Author Contributions

SM conceived the manuscript. SM and AR wrote the manuscript, critically revised it, and approved the final version.

### Conflict of Interest Statement

The authors declare that the research was conducted in the absence of any commercial or financial relationships that could be construed as a potential conflict of interest.
